# Percutaneous Coronary Intervention Mortality, Cost, Complications, and Disparities after Radiation Therapy: Artificial Intelligence-Augmented, Cost Effectiveness, and Computational Ethical Analysis

**DOI:** 10.3390/jcdd10110445

**Published:** 2023-10-30

**Authors:** Dominique J. Monlezun

**Affiliations:** 1 Department of Cardiology, The University of Texas M.D. Anderson Cancer Center, Houston, TX 77030, USA; dominique.monlezun@gmail.com or dominique.j.monlezun@uth.tmc.edu or ceo@gsasai.com; 2 Center for Artificial Intelligence & Health Equities, Global System Analytics & Structures (GSAS), New Orleans, LA 70112, USA

**Keywords:** cardio-oncology, radiation, PCI, artificial intelligence, cost, ethics, equity

## Abstract

The optimal cardio-oncology management of radiation therapy and its complications are unknown despite the high patient and societal costs. This study is the first known nationally representative, multi-year, artificial intelligence and propensity score-augmented causal clinical inference and computational ethical and policy analysis of percutaneous coronary intervention (PCI) mortality, cost, and disparities including by primary malignancy following radiation therapy. Bayesian Machine learning-augmented Propensity Score translational (BAM-PS) statistics were conducted in the 2016–2020 National Inpatient Sample. Of the 148,755,036 adult hospitalizations, 2,229,285 (1.50%) had a history of radiation therapy, of whom, 67,450 (3.00%) had an inpatient AMI, and of whom, 18,400 (28.69%) underwent PCI. Post-AMI mortality, costs, and complications were comparable with and without radiation across cancers in general and across the 30 primary malignancies tested, except for breast cancer, in which PCI significantly increased mortality (OR 3.70, 95%CI 1.10–12.43, *p* = 0.035). In addition to significant sex, race, and insurance disparities, significant regional disparities were associated with nearly 50 extra inpatient deaths and over USD 500 million lost. This large clinical, cost, and pluralistic ethical analysis suggests PCI when clinically indicated should be provided to patients regardless of sex, race, insurance, or region to generate significant improvements in population health, cost savings, and social equity.

## 1. Introduction

The growing co-incidence, life expectancy, and costs associated with cancer and cardiovascular disease accelerate the increased societal, public health, and clinical awareness and investment in their appropriate prevention, diagnosis, and treatment, especially as the global population ages and health resources shrink to address these top mortality causes [1,2]. Radiation therapy is a particularly prevalent treatment option in cancer (for nearly half of patients), though it carries long recognized cardiac risks with nearly 90% of radiation subjects ultimately diagnosed with coronary artery disease (CAD). Thoracic radiation in particular at sufficient doses increases fibrosis of all cardiac components (conduction system, coronaries, valves, myocardium, and pericardium) and thus the risk of arrythmias, CAD, valvopathy, cardiomyopathy, and pericardial disease. Prior to the 1970s, there was an over seven-fold increase in cardiovascular deaths after radiation therapy, while current radiation therapy appears to carry upwards of only a two-fold increased risk. Even with improved therapies, radiation’s cardiotoxicities and cardiac complications appear to persist longitudinally in a dose-dependent fashion starting within five years after exposure and lasts for over 20 years [3,4]. This includes independently increasing mortality after percutaneous coronary intervention (PCI), which is a mainstay of treatment for CAD, especially when it progresses to the point of causing symptomatic acute myocardial infarction (AMI). A 2022 meta-analysis spanning 13,941 patients with upwards of 16 years of follow-up demonstrated that radiation therapy was associated with a nearly 30% increased relative risk of all-cause mortality following PCI [2]. Yet, there is no clear consensus on the optimal selection of patients for PCI following radiation therapy nor a defined prevalence of procedural and population disparities undermining equitable population health, which is increasingly recognized as ethically problematic and economically costly to societies. Yet, prior studies have been limited by smaller sample sizes, geographic region, and a lack of primary malignancy and disparity analyses. This is therefore the first known nationally representative, multi-year, artificial intelligence and propensity score-augmented causal inference and computational ethical and policy analysis of post-radiation-therapy PCI mortality, cost, complications, and disparities including by primary malignancy.

## 2. Methods

### 2.1. Study Design

**Figure 1:**
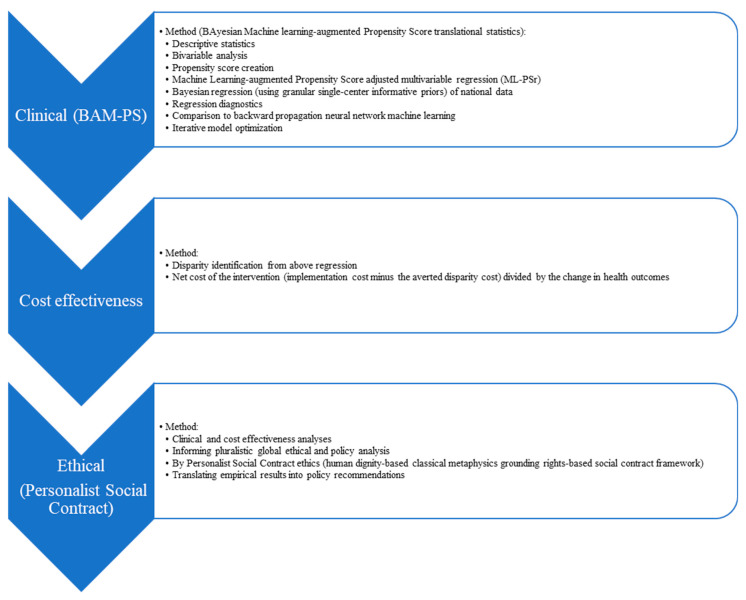
Study methodology: AI-driven Computational Ethics and policy analysis steps.

The 2016, 2017, 2018, 2019, and 2020 National Inpatient Sample (NIS) datasets were selected for this study as they are among the latest available datasets and the first to use ICD-10 coding and so better reflect current clinical trends in diagnoses, procedures, and outcomes compared to prior years. Study inclusion criteria included all NIS hospitalizations for adults aged 18 years or older during the above index time periods. This study utilized NIS data according to the ethical principles in the Declaration of Helsinki and the regulatory standards of the nation of origin (and thus did not require IRB review, exempt determination, or informed consent as confirmed by the United States National Bureau of Economic Research and the Department of Health and Human Services’ Agency for Healthcare Research and Quality [AHRQ]). Integrated comprehensive analysis detailed below was conducted using an AI-driven Computational Ethics and policy analysis (AiCE), including the first empirical step (BAM-PS detailed below, featuring a propensity score-based clinical analysis then a cost effectiveness analysis) followed by the second ethical step (using the Personalist Social Contract ethics framework to inform a computational ethical and policy analysis to advance the translation of the above results into equitable and effective health policy) (Figure 1).

### 2.2. Data Source

The data source for this study was the largest all-payer inpatient administrative dataset in the United States, the NIS, sponsored by the AHRQ and maintained within the Healthcare Cost and Utilization Project (HCUP). The NIS includes approximately 1 of every 5 hospital discharges from approximately 4500 hospitals. To reduce sampling bias, the sampling strategy has been modified in the most recent data to produce results more generalizable to all inpatient discharges in the country. The dataset includes demographic, comorbidity, procedural, mortality, length of stay, and cost for each adult hospitalization.

### 2.3. Descriptive and Bivariable Statistical Analysis

Descriptive statistics and bivariable analysis by prior radiation therapy was performed for the full sample and in sub-group analysis by year (2016, 2017, 2018, 2019, and 2020). Comorbidities were selected for analysis (and identified in the dataset by their ICD-10 codes) based on their clinical and/or statistical significance identified in prior published studies and current clinical practice. The comorbidities included in this study were hypertension (HTN), congestive heart failure (CHF), chronic obstructive pulmonary disease (COPD), chronic kidney disease (CKD), and metastases. For continuous variables, independent sample t tests were performed to compare means and Wilcoxon rank sum tests were performed for medians. For categorical variables, Pearson chi-squared tests or Fisher’s exact tests were performed to compare proportions as applicable.

### 2.4. Regression Statistical Analysis, Machine Learning Analysis, and Model Optimization

The primary outcome was mortality, and the secondary outcomes were prior radiation therapy, PCI, complications, and total hospitalization cost in (in U.S. dollars [USD]). To maximize the likelihood of valid (externally and internally) and replicable results, regression model performance was optimized according to the following sequential process. First, variables that were clinically or statistically significant were identified in the existing literature, clinic practice, and bivariable analysis to be considered in the final regression models. Second, those variables were included in forward and backward stepwise regression to augment decision making on the variables ultimately included in the final regression models. Third, the regression results were compared to those generated by backward propagation neural network machine learning to ensure comparability by root-mean-square error and accuracy. Fourth, regression model performance was additionally assessed with correlation matrix, area under the curve, Hosmer–Lemeshow goodness-of-fit test, Akaike and Schwarz Bayesian information criterion, variance inflation factor, and tolerance, multicollinearity, and specification error. Fifth, the models were re-run continually with fine tuning of the final models and variables until the above process confirmed that optimal performance was reached. Based on the above process, all regression models ultimately included age, sex, race, insurance, income, region, metastases, and mortality risk as calculated by the NIS according to the diagnosis-related group (DRG). Other variables were excluded based upon the machine learning analysis and diagnostic testing to produce the most clinically and statistically justifiable models.

### 2.5. Bayesian Machine Learning-Augmented Propensity Score Translational (BAM-PS) Statistics

Multivariable regression was then conducted using the above process within BAM-PS. This novel hybrid analytic methodology leverages the synergistic advantages of three methodological components by integrating them with each other: (a) ML-PSr (Machine Learning-augmented Propensity Score adjusted multivariable regression), in which the traditional statistical methodology of causal inference-based propensity score analysis is augmented (b) by machine learning capable of handling higher dimensional, more complex, and faster data streams, and then translates its results as informative priors for (c) Bayesian regression [5,6,7,8,9,10]. BAM-PS seeks to preserve the internal validity in analytic methodology while expanding it (i.e., by reducing the likelihood of relevant omitted variables) and its external validity (by increasing generalizability through a greater number of data sources to more accurately and precisely reflect real-world clinical practice, ultimately generating more timely, accurate, precise, and relevant predictions to augment organizational and clinical decision making in the AI-augmented and transforming healthcare systems). BAM-PS enables both direct (through integration) and indirect (through informative priors) linked datasets and data streams, including combining smaller, more granular datasets with larger, more generalizable datasets. This methodology thus is meant to further provide a more effective, efficient, and equitable analytics means that better approximates the real-time and more complex distributed cloud-fog-edge-based data collection, computing, and informed decision making in the emerging model of healthcare as an integrated global digital health ecosystem; this ecosystem leverages and integrates diverse partners (international institutions, healthcare systems, public health agencies, technology companies, governments, and community organizations, etc.) to optimize equitable value-based healthcare and societal wellbeing, particularly through AI-enabled Big Data and the Internet of Things, all within the global digital ecosystem generated by the Fourth Industrial Revolution [7,8]. Traditional statistics alone are therefore increasingly insufficient for the scope, speed, and complexity of the reality of modern healthcare, while AI alone lacks the broad understanding and acceptance of the health community. BAM-PS accordingly utilizes both domains of analytics together within the larger integrated AiCE, previously demonstrated for real-time clinical decision support through integration with electronic health records and other data streams along with health and organizational workflows.

Multivariable regression using this process was performed in two levels: first for the overall adult hospitalized sample to assess disparities stratified by year, and second, within acute myocardial infarction (AMI) stratified by year. In the sub-group regression, the separate outcomes of PCI, post-radiation therapy PCI, complications, costs, and mortality were assessed. For this study, the propensity score for the likelihood of receiving radiation therapy was first created (the treatment, utilizing the same above variables from the final regression model given the double propensity score adjustment method) [6,7,8], balance was confirmed among blocks, and then, the propensity score was included in the final regression models as an adjusted variable. Multivariable regression was then conducted for the above outcomes, with final model performance optimized using backward propagation neural networks. This process was completed using a sequential prospective cohort study of patients with cardiovascular disease and cancer in a large, single center, academic medical center in the southeastern United States featuring among the world’s first cardio-oncology departments. The process was then repeated in the NIS, using the above results as informative priors.

### 2.6. Cost Effectiveness Analysis

Cost-effectiveness analysis (CEA) was conducted according to the commonly accepted methodology described by the US Centers for Disease Control and Prevention (CDC): the net cost of the intervention (implementation cost minus the averted cost) divided by the change in health outcomes [11].

### 2.7. AI-Driven Computational Ethics and Policy Analysis

The second or ethical-policy step within AiCE was then conducted by integrating the above quantitative analyses with ethical analysis using the pluralistic global bioethical framework of the Personalist Social Contract (PSC) [7,8]. The PSC is a novel integration of a modern ethics framework (utilitarianism-informed Rawlsian social contract of political liberalism, bounded by Kantian deontology and informed by feminist, Marxist, deconstructionist, and ecological ethics) rooted in the metaphysical foundation of classical ethics (Thomistic–Aristotelian virtue ethics, articulated by William Carlo’s *esse*/essence revision of Norris Clarke’s Strong Thomistic Personalism, a derivative formulation of Thomism as a development of classical Aristotelianism). 

The PSC was chosen as the primary ethical framework for its (a) practical, (b) political, and (c) philosophical advantages over competing frameworks. (a) Practically, it is historically articulated in (and makes philosophically intelligible) the world’s most dominant and cited ethical system (of human dignity-based rights and duties) as expressed paradigmatically by the United Nation’s 1948 *Universal Declaration of Human Rights* (*UDHR*) and derivative systems of modern international law and related international ethical conventions. (b) Politically, it substantively accounts for and can facilitate the convergence of the world’s nations (including through the United Nations explicitly grounded in the *UDHR*) and belief systems on shared ethical conclusions as historically demonstrated (since the modern world united at the end of World War II to prevent future such catastrophic world wars and address other existential challenges). (c) Philosophically, it avoids the foundational metaphysical weaknesses (and resultant logical self-contradictions and struggles for deriving durable ethical conclusions within modern ethics) through the classical Aristotelian-derived Thomistic Personalism. In summary, the PSC argues that the world’s diverse belief systems (including Buddhist, Christian, Confucian, Daoist, folk, Hindu, Islamic, Jewish, and secular) converge not only existentially and substantively but also metaphysically and ethically in the shared conviction of the intrinsic and inviolable dignity of every human person. This dignity is most fully realized in the commitment to the common good, which in turn safeguards the good of each person as a rational dependent animal from our first to final moments: individual justice (giving to each what is due to them) at scale thus translates into (or at least begins to better approximate) collective peace. 

### 2.8. Model Validation, Reporting, and Analytic Software

Mean values are reported with standard deviation (SDs). Fully adjusted regression results were reported with 95% confidence intervals (CIs) with statistical significance set at a 2-tailed *p*-value of <0.05. Statistical analysis was performed with STATA 18.0 (STATACorp, College Station, TX, USA), and machine learning analysis was performed with Java 9 (Oracle, Redwood Chores, CA, USA). 

## 3. Results

### 3.1. Descriptive Statistics of the Overall Sample by Prior Radiation, AMI, PCI, Malignancy, and Year

Of the 148,755,036 adult hospitalizations from 2016 to 2020, 2,229,285 (1.50%) had a history of radiation therapy, of whom, 67,450 (3.00%) had an inpatient AMI, and of whom, 18,400 (28.69%) underwent PCI. The prevalence of post-radiation therapy AMI linearly increased during the study period from 2016 (2.58%) to 2017 (2.78%) to 2018 (2.94%) to 2019 (3.16%) to 2020 (3.54%). Meanwhile, the PCI prevalence linearly decreased from 2016 (47.28%) to 2017 (25.10%) to 2018 (24.02%) to 2019 (23.89%) to 2020 (23.15%). Of post-radiation therapy AMI hospitalizations receiving PCI from 2016 to 2018, the primary malignancies with a high prevalence included prostate (27.03%), breast (18.30%), lung (15.55%), head/neck (6.18%), and skin cancers (4.33%). 

### 3.2. Bivariable Analysis of Demographics, Comorbidities, and Outcomes by Prior Radiation and Year

In bivariable analysis from 2016 to 2020, having versus not having prior radiation had a significantly higher mean age (67.04 [SD 14.77] versus 51.18 [25.96]) and proportion of males (56.41% versus 50.25%), whites (74.60% versus 65.03%), Medicare (63.27% versus 41.68%), and highest income quartile (25.36% versus 19.68%), but also more HTN (57.99% versus 44.97%), CHF (6.38% versus 5.38%), COPD (22.57% versus 14.36%), complications (3.78% versus 2.11%), and unadjusted mortality (3.39% versus 2.32%), while having a significantly lower prevalence in lower likelihood of receiving inpatient PCI (1.46% versus 1.86%), African Americans (13.00% versus 15.40%), Hispanics (6.98% versus 12.40%), and Medicaid (9.41% versus 22.23%) (all *p* < 0.001) (Table 1). Having versus not having prior radiation therapy was associated with significantly higher total mean inpatient costs: USD 66,383.18 (87,497.64) versus USD 54,259.55 (103,574.23), *p* < 0.001. The direction and magnitude of these associations were generally stable each year from 2016 to 2020. 

### 3.3. Bivariable Analysis of AMI and Post-AMI PCI by Prior Radiation and Year

AMI was significantly less likely with versus without radiation history from 2016 to 2020 (1.16% versus 1.34, *p* < 0.001). Yet in AMI, PCI was significantly less likely to be carried out post-radiation (0.96 versus 1.26%, *p* < 0.001). The likelihood of receiving PCI progressively decreased post-radiation therapy from 2016 (0.81 versus 1.09%) to 2017 (0.82% versus 1.14%) to 2018 (0.92% versus 1.26%) to 2019 (1.08 versus 1.44%); by 2020, this trend began reversing (1.17% versus 1.38%) (all *p* < 0.001). 

### 3.4. Multivariable Regression of Radiation Therapy Disparities Stratified by Year

ML-PSr was conducted to identify and report below significant and independent predictors of radiation therapy, fully adjusted for demographics and clinical confounders. 

In 2016, Hispanics versus Caucasians were significantly less likely to receive radiation therapy (OR 0.91, 95%CI 0.88–0.94, *p* < 0.001), as were Medicaid (0.92, 95%CI 0.89–0.95, *p* < 0.001), VA (OR 0.85, 95%CI 0.80–0.90, *p* < 0.001), and uninsured patients (OR 0.8, 95%CI 0.76–0.88, *p* < 0.001) compared to those with commercial insurance. Compared to the lowest income quartile, the fourth (OR 1.24, 95%CI 1.21–1.27, *p* < 0.001) and third quartiles (OR 1.10, 95%CI 1.08–1.13, *p* < 0.001) were significantly more likely to receive it, amid significant geographic disparities. 

In 2017, Hispanics were less likely to receive radiation therapy (OR 0.94, 95%CI 0.91–0.97, *p* < 0.001) in addition to Asians (OR 0.94, 95%CI 0.90–0.99, *p* = 0.026), as were Medicaid (0.91, 95%CI 0.88–0.94, *p* < 0.001), VA (OR 0.90, 95%CI 0.85–0.96, *p* = 0.001), and uninsured patients (OR 0.79, 95%CI 0.73–0.85, *p* < 0.001). Compared to the lowest income quartile, the fourth (OR 1.23, 95%CI 1.20–1.26, *p* < 0.001) and third quartiles (OR 1.11, 95%CI 1.0–1.14, *p* < 0.001) were significantly more likely to receive it, amid significant geographic disparities.

In 2018, Hispanics were less likely to receive radiation therapy (OR 0.97, 95%CI 0.94–0.999; *p* = 0.041), as were Medicaid (0.92, 95%CI 0.89–0.95; *p* < 0.001), VA (OR 0.91, 95%CI 0.86–0.96, *p* < 0.001), and uninsured patients (OR 0.82, 95%CI 0.77–0.88, *p* < 0.001); meanwhile, compared to the lowest income quartile, the fourth (OR 1.18, 95%CI 1.16–1.21, *p* < 0.001) and third quartiles (OR 1.08, 95%CI 1.05–1.10, *p* < 0.001) were significantly more likely to receive it, amid significant geographic disparities.

In 2019, Medicaid (0.90, 95%CI 0.87–0.92, *p* < 0.001), VA (OR 0.90, 95%CI 0.86–0.95, *p* < 0.001), and uninsured patients (OR 0.81, 95%CI 0.76–0.86, *p* < 0.001) were less likely to receive radiation therapy; meanwhile, compared to the lowest income quartile, the fourth (OR 1.17, 95%CI 1.15–1.20, *p* < 0.001) and third quartiles (OR 1.10, 95%CI 1.08–1.12, *p* < 0.001) were significantly more likely to receive it with significant geographic disparities. 

Finally, in 2020, Native Americans were less likely to receive radiation therapy (OR 0.76, 95%CI 0.67–0.86, *p* < 0.001), as were Medicaid (0.90, 95%CI 0.87–0.93, *p* < 0.001), VA (OR 0.92, 95%CI 0.87–0.97, *p* = 0.001), and uninsured patients (OR 0.78, 95%CI 0.73–0.83, *p* < 0.001). Compared to the lowest income quartile, the fourth (OR 1.19, 95%CI 1.16–1.21, *p* < 0.001) and third quartiles (OR 1.11, 95%CI 1.09–1.14, *p* < 0.001) were significantly more likely to receive it, amid significant geographic disparities.

### 3.5. Multivariable Regression of PCI in AMI Stratified by Year

In ML-PSr fully adjusted for demographics and clinical confounders, AMI hospitalizations following radiation therapy did not significantly predict the odds of receiving PCI in any year, including 2016 (OR 1.10, 95%CI 0.99–1.22, *p* = 0.070), 2017 (OR 1.00, 95%CI 0.90–1.11, *p* = 0.986), 2018 (OR 0.98, 95%CI 0.89–1.09, p = 0.745), 2019 (OR 0.97, 95%CI 0.89–1.06, *p* = 0.532), and 2020 (OR 1.01, 95%CI 0.92–1.11, *p* = 0.826).

### 3.6. Multivariable Regression of Post-Radiation Therapy PCI in AMI Stratified by Year

**Figure 2:**
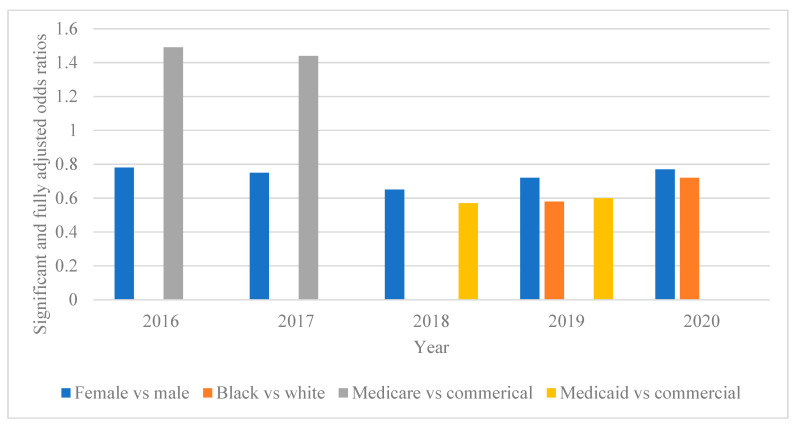
Machine Learning-augmented Propensity Score adjusted multivariable regression of post-radiation PCI in AMI stratified by year (all *p* < 0.001).

Despite fully adjusted for clinical confounders, females versus males were significantly less likely to receive PCI across all five years, while in 2018–2019, Medicaid versus commercial and, in 2019–2020, African Americans versus Caucasians were significantly less likely to receive PCIs (Figure 2). The greatest disparities were in 2018, specifically for females (OR 0.65, 95%CI 0.52–0.80, *p* < 0.001) and Medicaid (OR 0.57, 95%CI 0.34–0.96, *p* = 0.036). There were no other significant and independent disparities. 

### 3.7. Multivariable Regression of Radiation Therapy on Post-PCI Complications in AMI Stratified by Year

Overall complications were assessed as a composite measure of post-PCI acute respiratory failure, hemorrhage, vascular complications (including vessel puncture), cardiac complications (including acute heart failure, pericardial effusion, heart block, and cardiac arrest), acute kidney injury (AKI), cerebrovascular accident (CVA), pulmonary embolus (PE), and infection. After fully adjusting for clinical confounders, radiation therapy only significantly increased the odds of post-PCI PE in 2017 (OR 2.57, 95%CI 1.02–6.49, *p* = 0.045), but no other complications in any other years (Table 2).

### 3.8. Multivariable Regression of Total AMI Hospitalization Costs with PCI Stratified by Year

Radiation therapy did not significantly increase costs in any year when AMI was treated via PCI: in 2016 (USD−5325.87, 95%CI −10,941.66–289.92, *p* = 0.063), 2017 (USD−1786.61, 95%CI −8781.15–5207.93, *p* = 0.617), 2018 (USD−2480.36, 95%CI −9665.75–4,705.02, *p* = 0.499), 2019 (USD−6735.40, 95%CI −13,825.11–354.31, *p* = 0.063), or 2020 (USD-2941.00, 95%CI −10,551.44–4669.45, *p* = 0.449).

### 3.9. Multivariable Regression of Radiation Therapy on Post-PCI Mortality in AMI Stratified by Year

After fully adjusting for demographics and clinical confounders, radiation therapy was associated with significantly reduced post-PCI mortality odds in 2016 (OR 0.60, 95%CI 0.40–0.91, *p* = 0.017), 2018 (OR 0.56, 95%CI 0.32–0.95, *p* = 0.031), and 2019 (OR 0.51, 95%CI 0.31–0.84, *p* = 0.007), but not in 2017 or 2020. Stratified by prior radiation, post-AMI PCI significantly reduced mortality more in patients with versus without radiation therapy in 2016 (OR 0.44 versus 0.58) and 2019 (OR 0.58 versus 0.99), but not overall from 2016 to 2020 (Table 3). 

### 3.10. Multivariable Regression of Radiation Therapy on Post-PCI Mortality in AMI Stratified by Year and Primary Malignancy

After fully adjusting for demographics and clinical confounders, radiation therapy was not significantly associated with post-PCI mortality odds in the top primary malignancies noted above, including prostate, breast, lung, head/neck, and skin. But when mortality was analyzed among post-radiation therapy AMI hospitalizations within each of the above primaries, PCI only significantly increased mortality in breast cancer (OR 3.70, 95%CI 1.10–12.43, *p* = 0.035).

### 3.11. Cost Effectiveness Analysis

The 2020 data were utilized for AiCE calculations since they are the most recent data that therefore best reflect the current clinical practice. In post-radiation-therapy AMI fully adjusted for demographics and clinical confounders, those in the Mid-Atlantic (OR 0.62, 95%CI 0.44–0.87, *p* = 0.005) were significantly less likely compared to those in the South Atlantic to receive PCI. The Mid-Atlantic region consisted of New York, Pennsylvania, and New Jersey. The South Atlantic region consisted of Delaware, Maryland, the District of Columbia, Virginia, West Virginia, North Carolina, South Carolina, Georgia, and Florida. The South Atlantic region was chosen as the base region as it had the highest percentage of PCI for AMI among patients with prior radiation in 2020 (at 21.09%). Compared to the South Atlantic, according to the difference in the population-averaged predictive margins, the Mid-Atlantic had a 6.39% significantly lower likelihood of receiving PCI among AMI hospitalizations with prior radiation. In the overall adult population nationally, PCI in AMI significantly reduced mortality (OR 0.89, 95%CI 0.85–0.93, *p* < 0.001) with a 0.86% significantly decreased likelihood of inpatient mortality according to population-averaged predictive margins. Therefore across the 5 years, there were an additional estimated 45 inpatient deaths or USD 522.50 million lost in the Mid-Atlantic versus South Atlantic AMI hospitalizations following radiation therapy. Among post-radiation AMI, PCI significantly increased total hospitalization costs by USD 61,049.82 (95%CI 54,063.22–68,036.43, *p* < 0.001). According to the Centers for Disease Control approach, cost effectiveness was calculated as the difference between total hospitalization costs of PCI minus the total statistical value of lives [calculated by the 2020 United States federal government as reported by the Department of Transportation] saved in post-radiation-therapy AMI receiving PCI divided by the number of additional lives saved by PCI. This equaled USD 11,525,085 saved per AMI hospitalization with PCI. 

### 3.12. AI-Driven Computational Ethics and Policy Analysis

When the above empirical results were applied to this concrete ethical situation, the formal PSC argument is as follows. (Premise 1) There appear to be significant regional disparities for post-radiation-therapy AMI patients receiving the PCI standard of care significantly less often in the Mid-Atlantic versus the South Atlantic that are not sufficiently explained by clinical factors; in addition, there appears to be significant racial and insurance disparities associated with Hispanic, Medicaid, and uninsured patients receiving radiation therapy in the first place. (Premise 2) Life and equal societal protection are fundamental and globally recognized individual and state rights. (Premise 3) Respect for dignity at the individual level requires respecting the person’s rights to goods (beginning with the primary good of life) necessary for the person to develop through a just and stable commitment to the common good and thus the community in reciprocal care for the individual. (Premise 4) Respect for dignity at the communal level requires respecting other cultures as the communal manifestations of their constitutive individuals seeking through justice. (Premise 5) Social disparities in medical care can produce disproportionate mortality and morbidity burdens in communities, resulting in a disproportionate threat to the preservation of those persons and related cultures, and thus the impoverishment of the global human community. (Premise 6) The reduction of such disparities through clinically indicated PCI alone may result in dozens of lives saved along with hundreds of millions of dollars. (Premise 7) Continued disparities in the standard of care for cancer and cardiac disease undermine respect for the rights of patients and respect for their cultures, which is critical to the wellbeing of the global human family that encompass all peoples and cultures. (Conclusion) Therefore, clinical, economic, and ethical justification supports greater healthcare policy, healthcare system, and public health investment reducing such disparities for the good of such patients, communities, and the wider public.

## 4. Discussion

This is the first known multi-year nationally representative machine learning and propensity score-augmented analysis of AMI, PCI, and its disparities along with mortality and cost outcomes based on the presence or absence of prior radiation therapy, primary malignancy, and year. This study across 5 years and nearly 150 million hospitalizations identified over 2.23 million annual hospitalizations following radiation therapy, of which 67,000 had AMI (with the most prevalent primary malignancies in post-radiation AMI were prostate, breast, lung, head/neck, and skin cancer). A longitudinal unadjusted analysis suggested that AMI increased while PCI decreased following radiation therapy progressively every year from 2016 to 2020 (as it appears such AMI was increasingly treated pharmacologically instead of procedurally). Unlike prior emblematic studies on this topic [1,2,3,4], this study provides novel evidence of these time trends, while also showing the nationally representative clinical profile of post-radiation therapy patients, which appears to show that they generally are more likely to be older, male, Caucasian, and richer compared to non-radiation patients, while the former have more HTN, COPD, complications, and unadjusted mortality—though AMI was less likely among them (as was receiving a PCI when presenting to the hospital with AMI as noted above). Hispanics, lower income, and non-commercially insured patients generally appeared to be less likely to receive radiation therapy, even after adjusting for clinical severity and comorbidities. 

**Figure 3:**
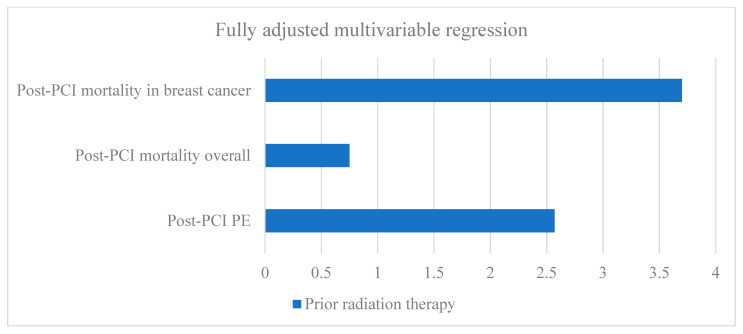
Central illustration of study highlights 67,450 acute myocardial infarction hospitalizations, 2016–2020. PCI, percutaneous coronary intervention; PE, pulmonary embolus; fully adjusted for clinical confounders and demographics.

Aside from these disparities in receiving radiation therapy in the first place, this study’s adjusted regression analysis suggests that among radiation therapy patients, those who experienced AMI hospitalizations were significantly less likely to receive PCI if they were female, across all five years (along with transient disparities for African Americans and those with Medicaid insurance). Across all five years, complications and costs were generally comparable—while mortality was potentially even lower—with versus without radiation therapy history. Study highlights on mortality are graphically featured in Figure 3. Additionally, there were significant regional disparities in PCI in post-radiation AMI hospitalizations (even controlling for clinical confounders and severity), specifically in the Mid-Atlantic versus South Atlantic, which may result throughout the study period of nearly 50 extra inpatient deaths and over USD 500 million lost. Correcting such disparities may result in over USD 11.53 million per life saved. Additionally, there were significant disparities in Hispanic, Medicaid, and uninsured patients receiving radiation therapy in the first place not sufficiently explained by clinical factors. Computational ethical and economic analyses support the reversal of such disparities for the cost savings and the promotion of justice to respect individual dignity and cultural diversity critical for the global human family.

In contrast to the above studies [1,2,3,4], this study suggests that though radiation therapy is a known mortality risk factor including after PCI, it should not in and of itself deter clinicians from providing PCI when clinically indicated and appropriate for AMI, especially as it appears safe and not excessively costly for patients with and without a history of radiation therapy. When there is not equitable care among such patients, the economic costs and unethical injustice are prohibitive to allow such disparities to persist. There continues to be rigorous clinical and research advances in reducing cardiotoxicity from radiation therapy (including more precise radiation doses, fields, and methods—including proton therapy and intensity-modulated radiation therapy—in addition to enhanced risk stratification by tumor genetics, patient genetics, and pre-existing cardiovascular disease, coupled with post-radiation therapy monitoring with more complex cardio-oncology multi-disciplinary teams managing the above process). This study provides additional data-driven insights to scale such individual level advances to the population and potentially even global levels.

This study has a number of novel advantages compared to past studies. It is nearly as long as the leading longitudinal cohort *JAMA* study by Reed et al. 2016, while including 18,400 post-radiation therapy PCI hospitalizations compared to the 314 subjects in the above study [4]. It is larger still than the 13,941 subjects in the Thakker et al. 2022 analysis, the most recent meta-analysis on this subject which spanned only four studies as of the writing of this paper [2], while this analysis drew from nearly 4,500 hospitals in the nationally representative NIS dataset to provide greater generalizability and internal validity. This study would additionally be the first study separately to include post-radiation therapy PCI disparity, cost, AI, propensity score, cost effectiveness, and computational ethical analyses. Finally, this study provides robust evidence for the current state of the art, advancing a more precise risk stratification for post-radiation therapy patients presenting with AMI and being considered for PCI. A 2019 Cleveland Clinic analysis with a mean follow-up of 5.4 years suggested that major adverse cardiovascular and cerebrovascular events were more likely in patients with a SYNTAX score of at least 11, CKD3-5, and CHF with a New York Heart Association class of at least 3 [12]. Such factors were not found in this national analysis to be significant predictors of adverse events, at least in the index hospitalization across cancers in general. However, this study does suggest that within the primary malignancies analyzed separately, inpatient PCI when performed during AMI hospitalizations in breast cancer does significantly increase the odds of inpatient death by nearly four-fold. 

This study has a number of notable limitations as well, supporting caution when interpreting the findings. The study’s non-randomized observational study design utilized administrative short-term inpatient data only collected from the United States. To mitigate the related biases that these limitations can introduce to the study, it utilized a multi-year nationally representative sample from thousands of hospitals, in addition to a robust causal inference statistical methodology that featured a robust, comprehensive methodology that emphasized extensive model optimization techniques that have been published in over a dozen separate cardiology studies (included those referenced in the methods section).

## 5. Conclusions

The results from the largest, longest, and most complete nationally representative study of post-radiation therapy AMI and PCI suggest that significant disparities persist in radiation therapy and PCI following it that are not sufficiently explained by clinical factors, but reversing such disparities could significantly improve patient outcomes and be more cost effective and societally equitable for population health. 

## Figures and Tables

**Table 1:** Bivariable analysis of adult hospitalizations from 2016 to 2020 by history of radiation (all *p* < 0.001).

Variable, (%)	2016–2020		2016		2017		2018		2019		2020	
	Without XRT	With XRT	Without XRT	With XRT	Without XRT	With XRT	Without XRT	With XRT	Without XRT	With XRT	Without XRT	With XRT
Age, mean (SD)	51.18 (25.96)	67.04 (14.77)	57.39 (20.37)	67.17 (13.75)	49.37 (27.43)	66.72 (15.17)	49.65 (27.39)	66.91 (15.10)	49.89 (27.38)	67.26 (14.83)	49.60 (27.24)	67.15 (15.01)
Female	56.41	50.25	58.25	50.26	56.47	50.33	56.18	50.55	55.91	50.29	55.24	49.80
Race												
White	65.03	74.60	67.70	75.45	64.74	74.74	64.49	74.31	64.78	74.43	63.46	74.05
Black	15.40	13.00	15.16	12.80	15.35	13.05	15.19	13.18	15.43	12.98	15.86	13.00
Hispanic	12.40	6.98	10.89	6.22	12.56	6.68	12.90	7.18	12.46	7.27	13.17	7.55
Asian	3.04	2.72	2.69	2.63	3.12	2.62	3.13	2.70	3.13	2.76	3.13	2.87
Native American	0.68	0.38	0.62	0.39	0.65	0.39	0.67	0.38	0.71	0.41	0.74	0.33
Other	3.45	2.32	2.93	2.51	3.58	2.51	3.61	2.24	3.50	2.15	3.63	2.20
Insurance												
Commercial	29.00	23.80	27.67	24.69	29.39	24.07	29.22	23.45	29.15	23.20	29.57	23.58
Medicare	41.68	63.27	46.72	62.71	40.47	63.07	40.80	63.52	40.86	63.84	39.55	63.21
Medicaid	22.23	9.41	18.74	9.25	23.37	9.54	22.98	9.60	22.68	9.31	23.38	9.35
VA	2.94	2.18	2.96	2.07	2.81	2.08	2.81	2.08	2.94	2.21	3.18	2.47
Uninsured	4.15	1.34	3.91	1.27	3.96	1.24	4.20	1.35	4.37	1.44	4.32	1.39
Income quartile												
Lowest	30.35	24.50	30.94	24.66	30.40	24.49	29.54	24.35	30.48	24.73	30.40	24.29
Second	26.30	24.61	25.64	23.66	26.47	24.41	26.93	25.06	25.32	23.89	27.12	26.03
Third	23.68	25.52	23.80	25.42	23.48	25.40	23.82	25.54	24.36	26.36	22.93	24.88
Highest	19.68	25.36	19.62	26.25	19.66	25.71	19.71	25.05	19.83	25.01	19.56	24.80
HTN	44.97	57.99	54.22	62.86	41.79	56.36	41.72	55.73	42.14	57.01	42.14	57.36
CHF	5.38	6.38	5.53	5.83	5.34	6.41	6.05	7.47	6.61	8.16	3.36	4.03
COPD	14.36	22.57	16.24	22.59	14.03	22.47	14.10	22.98	14.16	22.92	13.26	21.87
CKD3–5	10.90	11.90	11.36	10.73	10.28	11.59	10.91	12.30	11.66	13.47	10.27	11.39
Metastasis	2.78	25.76	3.01	25.86	2.63	25.58	2.69	25.92	2.76	25.76	2.80	25.70
Complications	2.11	3.78	3.98	6.07	1.47	3.23	1.49	3.26	1.95	3.13	1.65	3.20

**Table 2:** Machine Learning-augmented Propensity Score-adjusted multivariable regression of radiation on post-PCI complications in AMI stratified by year *.

Year	Hemorrhage	Cardiac	CVA	PE	Overall Complications
2016	OR 0.55, 95%CI 0.23–1.33, *p* = 0.183	OR 1.02, 95%CI 0.60–1.73, *p* = 0.952	OR 1.50, 95%CI 0.37–6.14, *p* = 0.570	OR 0.89, 95%CI 0.36–2.17, *p* = 0.793	OR 0.84, 95%CI 0.61–1.18, *p* = 0.320
2017	OR 1.00, 95%CI 1.00–1.00, *p* > 0.05	OR 0.20, 95%CI 0.03–1.46, *p* = 0.113	OR 1.35, 95%CI 0.18–9.91, *p* = 0.769	OR 2.57, 95%CI 1.02–6.49, *p* = 0.045	OR 0.80, 95%CI 0.39–1.63, *p* = 0.539
2018	OR 0.73, 95%CI 0.10-5.23, *p* = 0.752)	OR 0.61, 95%CI 0.20-1.93, *p* = 0.404	OR 1.43, 95%CI 0.20–10.40, *p* = 0.724	OR 1.14, 95%CI 0.36–3.65, *p* = 0.821	OR 0.68, 95%CI 0.32–1.45, *p* = 0.315
2019	OR 0.95, 95%CI 0.23–3.85, *p* = 0.941)	OR 1.52, 95%CI 0.81–2.89, *p* = 0.195	OR 0.83, 95%CI 0.12–6.00, *p* = 0.855	OR 0.60, 95%CI 0.15–2.48, *p* = 0.483	OR 0.1.03, 95%CI 0.63–1.69, *p* = 0.902
2020	OR 0.59, 95%CI 0.08–4.2, *p* = 0.602)	OR 0.82, 95%CI 0.30–2.23, *p* = 0.702	OR 1.02, 95%CI 0.14–7.41, *p* = 0.987	OR 0.62, 95%CI 0.15–2.63, *p* = 0.524	OR 0.70, 95%CI 0.37–1.32, *p* = 0.274

* PCI, percutaneous coronary intervention; AMI, acute myocardial infarction; CVA, cerebrovascular accident; PE, pulmonary embolus; OR, odds ratio; CI, confidence interval.

**Table 3:** Machine Learning-augmented Propensity Score-adjusted multivariable regression of radiation on post-PCI mortality in AMI stratified by year *.

Year	PCI in Patients without Radiation	PCI in Patients with Radiation
2016	OR 0.58, 95%CI 0.55–0.60, *p* < 0.001	OR 0.44, 95%CI 0.27–0.72, *p* = 0.001
2017	OR 0.84, 95%CI 0.80–0.88, *p* < 0.001	OR 0.88, 95%CI 0.53–1.45, *p* = 0.615
2018	OR 0.91, 95%CI 0.87–0.96, *p* < 0.001	OR 0.70, 95%CI 0.39–1.25, *p* = 0.225
2019	OR 0.99, 95%CI 0.94–1.04, *p* = 0.703	OR 0.58, 95%CI 0.35–0.97, *p* = 0.040
2020	OR 0.89, 95%CI 0.85–0.93, *p* < 0.001	OR 1.14, 95%CI 0.72–1.79, *p* = 0.577
2016–2020	OR 0.84, 95%CI 0.80–0.88, *p* = 0.104	OR 0.75, 95%CI 0.45–1.24, *p* = 0.290

* PCI, percutaneous coronary intervention; AMI, acute myocardial infarction; OR, odds ratio; CI, confidence interval.

## Data Availability

The data source for this study was the largest all-payer inpatient administrative dataset in the United States, the NIS, sponsored by the AHRQ and maintained within the Healthcare Cost and Utilization Project (HCUP).
